# Immunogenicity of an AS01-adjuvanted respiratory syncytial virus prefusion F (RSVPreF3) vaccine in animal models

**DOI:** 10.1038/s41541-023-00729-4

**Published:** 2023-09-29

**Authors:** Badiaa Bouzya, Ronan Nicolas Rouxel, Lionel Sacconnay, Romuald Mascolo, Laurence Nols, Stéphanie Quique, Loïc François, Anne Atas, Lucile Warter, Nancy Dezutter, Clarisse Lorin

**Affiliations:** 1 GSK, Rue de l’Institut 89, 1330 Rixensart, Belgium; 2 Akkodis, Belgium c/o GSK, Rue de l’Institut 89, 1330 Rixensart, Belgium; 3 Present Address: MSD Animal Health, Thormøhlensgate 55, 5006 Bergen, Norway

**Keywords:** Protein vaccines, Viral infection

## Abstract

Respiratory syncytial virus (RSV) causes a high disease burden in older adults. An effective vaccine for this RSV-primed population may need to boost/elicit robust RSV-neutralizing antibody responses and recall/induce RSV-specific T cell responses. To inform the selection of the vaccine formulation for older adults, RSVPreF3 (RSV fusion glycoprotein engineered to maintain the prefusion conformation) with/without AS01 adjuvant was evaluated in mice and bovine RSV infection-primed cattle. In mice, RSVPreF3/AS01 elicited robust RSV-A/B-specific neutralization titers and RSV F-specific polyfunctional CD4^+^ T cell responses exceeding those induced by non-adjuvanted RSVPreF3. In primed bovines, RSVPreF3/AS01 tended to induce higher pre-/post-vaccination fold-increases in RSV-A/B-specific neutralization titers relative to non-adjuvanted and Alum-adjuvanted RSVPreF3 formulations, and elicited higher RSV F-specific CD4^+^ T cell frequencies relative to the non-adjuvanted vaccine. Though AS01 adjuvanticity varied by animal species and priming status, RSVPreF3/AS01 elicited/boosted RSV-A/B-specific neutralization titers and RSV F-specific CD4^+^ T cell responses in both animal models, which supported its further clinical evaluation as prophylactic candidate vaccine for older adults.

## Introduction

Respiratory syncytial virus (RSV) is a leading cause of severe lower respiratory tract diseases (LRTD) in infants, and a significant cause of severe disease in adults with chronic medical conditions and older adults (aged ≥60 years; OA)^1–3^. The large unmet medical need and lack of a licensed prophylactic vaccine provide the impetus to develop vaccines for populations at increased risk of severe RSV disease^4^. Recently, the burden of RSV-associated acute respiratory infection in OA in high-income countries was estimated at circa 5.2 million cases, including 470,000 hospitalizations and 33,000 in-hospital deaths (in 2019)^5^. Moreover, the morbidity and mortality related to RSV are both similar—and in some years even more severe—as compared to the influenza-associated figures, especially in OA even though the vast majority of this population has been vaccinated against influenza^6,7^. Virtually all adults have been primed by RSV reinfections occurring throughout life, but the immunity derived from this priming progressively decreases in OA due to immunosenescence (age-related immune dysregulation)^8^. Definite immune correlates of protection have yet to be identified. While robust RSV neutralizing antibody (NAb) titers inhibit viral replication and prevent primary infection, they inconsistently prevent disease progression in break-through cases^1,8^. Cell-mediated immune (CMI) responses are thought to contribute to viral clearance, based on the link between pulmonary responses of cytotoxic CD8^+^ T lymphocytes (CTLs) and reduced disease severity^9^. Furthermore, T-helper 1 (Th1)-polarized CD4^+^ T cell responses play a role in RSV-infection control by supporting CTL development and improving humoral response quantity/quality^8^.

Immunosenescence changes the magnitude, composition, and functionality of innate and adaptive CMI responses^10,11^. With age, naive and IFN-γ–secreting RSV-specific T cells become less abundant and less functional and the formation of naive B cells from bone marrow declines, underpinning the increased susceptibility to viral infection faced by OA^8,10–15^. An effective vaccine for this population should thus be able to robustly recall/elicit humoral and CMI responses, by boosting/inducing strong and persistent NAb responses and restoring/inducing RSV-specific T cell responses^8^. Vaccine development focuses mainly on the highly conserved RSV fusion (F) glycoprotein, which is critical in the pathogenesis of RSV-associated LRTD^1,8^. The protein occurs naturally in a metastable prefusion (PreF) or a stable post-fusion (PostF) conformation. Among RSV NAbs, PreF-specific NAbs are the most effective in preventing infection^16–18^. In particular, NAbs targeting the PreF-exclusive antigenic site Ø, such as the D25 antibody, are highly potent and account for one-third of the total potency in human sera^16,18^. This may explain why several non-PreF–stabilized vaccine candidates failed to meet their primary endpoints in clinical efficacy trials^1^. PreF-stabilized subunit antigens are therefore considered a promising avenue for vaccine development^1,19,20^. Here, we report the preclinical evaluation of different formulations of a recombinant subunit protein that has been engineered to preferentially maintain a PreF conformation (RSVPreF3^21^).

In the context of an RSV-primed OA population, it may be beneficial to combine a stable PreF antigen with an adjuvant. In young adults, immune responses elicited by RSV PreF-based vaccines were comparable between formulations with or without aluminum salt (Alum)^22–24^. Adjuvant System (AS)01 is a liposome-based adjuvant, which contains the toll-like receptor (TLR)4 agonist 3-*O*-desacyl-4′-monophosphoryl lipid A (MPL) and the saponin QS-21^25,26^. These immunostimulants have been shown to act synergistically to promote an innate immune response profile associated with the induction of potent antigen-specific Th1-biased CD4^+^ T cell response and antibody responses^27–32^. AS01 is incorporated into licensed or candidate subunit vaccines^25,26^, including the licensed herpes zoster (HZ) vaccine for OA (containing varicella zoster virus glycoprotein E; VZV gE)^33^. A formulation of VZV gE with AS01_B_ elicited robust and persistent gE-specific CD4^+^ T cell and antibody responses, translating into high-level efficacy and immune memory responses persisting up to 10 years post-vaccination in OA^33,34^.

As the nature of vaccine-elicited immune responses can be both species- and antigen-specific^35,36^, we first assessed the capacity of AS01 to elicit and boost the RSV PreF-specific immune response in mice, and then consolidated our findings by comparing the abilities of RSVPreF3 formulated either with AS01, with Alum or without adjuvant, to boost baseline immunity in bovine (b)RSV pre-exposed bovines. The latter have emerged as a surrogate model of RSV-primed humans due to the high-level (~81%) sequence identity shared by bRSV and human (h)RSV F proteins^20,37^. The immune profiles detected here in both models suggest that RSVPreF3 formulations with AS01 could induce/boost CD4^+^ T cell responses and RSV-A/B-specific neutralization titers in OA, supporting the evaluation of AS01-adjuvanted RSVPreF3-based vaccine candidates in a subsequent clinical trial^21^.

## Results

### In RSV-naive mice, AS01 enhances functional antibody responses to RSVPreF3-based formulations in a dose-independent manner

We assessed the capacity of RSVPreF3/AS01 to elicit and boost RSV-specific immunity in mice, and evaluated the effect of the adjuvant by comparing the responses elicited by AS01-adjuvanted *vs* non-adjuvanted RSVPreF3 vaccines. AS01 was used at three different dose-levels to select the most immunogenic adjuvant-antigen ratio amongst the ratios tested. In each group, animals received three injections, two weeks apart (at D0, D14, D28) of either the same RSVPreF3 formulation across the three immunizations, or placebo (saline). Humoral and T cell responses in sera and spleens, respectively, were assessed at two weeks post-dose 2 (D28) and two weeks post-dose 3 (D42).

**Fig. 1:**
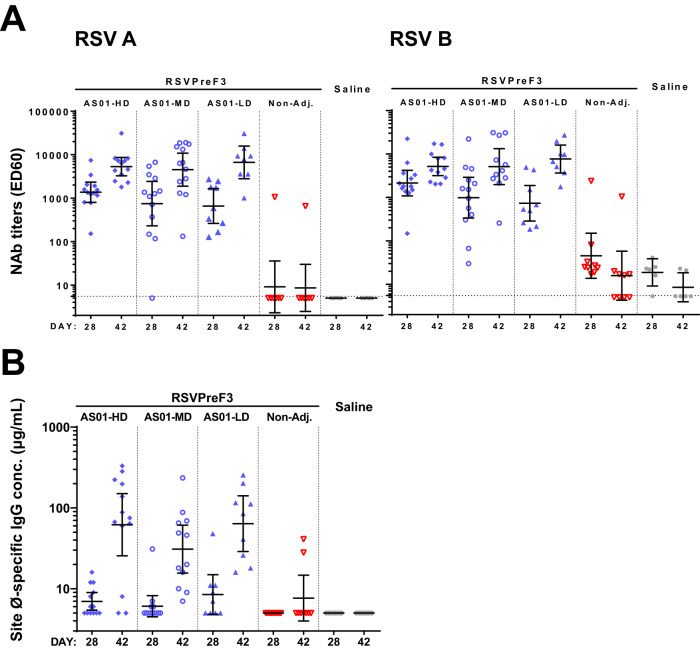
Antibody responses in mice. Naive CB6F1 mice received three injections two weeks apart (Days 0, 14, 28). They were administered AS01-adjuvanted RSVPreF3 vaccine (formulated with high-dose [HD], medium-dose [MD] or low-dose [LD] AS01; *n* = 13, 13 or 9, respectively), non-adjuvanted (Non-Adj.) RSVPreF3 vaccine (*n* = 9), or saline only (*n* = 6). Serum antibody titers were measured 14 days after doses 2 and 3 (Days 28 and 42, respectively). Data are presented as geometric mean titers with 95% confidence intervals (bars) and individual titers (symbols). **A** RSV-A/RSV-B neutralization titers (left/right panels) were obtained with RSV strains A Long and B 18537, respectively, and expressed as reciprocals of the serum dilution neutralizing 60% of virus (ED60). RSV-A/RSV-B neutralization titers in each AS01 group were significantly higher (*P* < 0.01; ANOVA for repeated measures) relative to the non-adjuvanted vaccine group, at both timepoints. Horizontal dotted lines represent the detection limit. **B** Site Ø-specific immunoglobulin (Ig)G antibody concentrations (conc.) were measured by D25-competition ELISA.

We first tested the ability of AS01 to enhance NAb responses against the prototypic RSV-A Long and RSV-B 18537 strains (Fig. 1A). In the three AS01 groups, the RSV-A/B-specific geometric mean neutralization titers (neutralization GMTs) detected after the second dose were increased after the third dose (D28 and D42 neutralization GMTs across AS01 groups: 659–1368 and 4527–8261 ED60, respectively). At both timepoints, RSV-A/B-specific neutralization GMTs were significantly higher in the AS01 arms *vs* the non-adjuvanted vaccine arm (*P* < 0.01; ANOVA for repeated measures), and similar across the AS01 arms. Titers were overall comparable between both RSV subtypes, as expected given that RSV F is well conserved^38^.

The significantly higher responses found for RSVPreF3 vaccines with *vs* without AS01 were corroborated by data from a similarly designed experiment, in which the immunogenicity of RSVPreF3 was compared to that of an RSV PostF antigen (Supplementary Fig. 1). This data also showed that for the AS01-adjuvanted formulations, the RSV-A neutralization GMTs post-dose 3 were significantly higher (*P* < 0.00001; one-way ANOVA with heterogeneous variance) for RSVPreF3 than for RSV PostF.

Because site Ø-specific antibody response magnitudes correlate with neutralizing activity^16^, we then used D25-competition ELISA to probe whether the observed antibody activity was directed to this PreF-exclusive site^18^. The human antibody D25 binds specifically to site Ø, therefore the presence of D25-competing antibodies informs of the antigen’s stability in the PreF conformation post-immunization. After the second dose, animals in the AS01 groups displayed low anti-site-Ø immunoglobulin (Ig)G responses (D28 geometric mean concentrations [GMCs] <10 EU/mL; Fig. 1B). This contrasted with the already robust neutralization titers at this timepoint, an effect which may be due to a difference in assay sensitivity. The majority of animals (32/34) responded after the third dose (D42), which distinctly increased the GMCs (by 9-fold, 5-fold, and 8-fold for high, medium and low-dose AS01, respectively). Conversely, only 2/9 of recipients of the non-adjuvanted vaccine responded at D42.

Thus, within the dose-range evaluated, AS01 had a potent, dose-independent effect not only on the magnitude, but also on the functionality of the humoral response, by promoting responses to a highly neutralization-sensitive epitope. This reflects preservation of the antigen in the more immunogenic PreF conformation after vaccination, and suggests the presence of CD4^+^ T cell help.

### AS01-adjuvanted RSVPreF3 elicits polyfunctional CD4^+^ T cell responses in RSV-naive mice

Given the reduced T cell immunity observed in OA^10,11^, we next investigated whether formulating RSVPreF3 with AS01 could also elicit RSV F-specific T cell responses in mice. Th1 cytokine (IL-2/IFN-γ/TNF-α)-expressing RSV F-specific CD4^+^ and CD8^+^ T cell frequencies were evaluated using intracellular cytokine staining (ICS) and flow cytometry (see Supplementary Fig. 2 for the gating strategy).

**Fig. 2:**
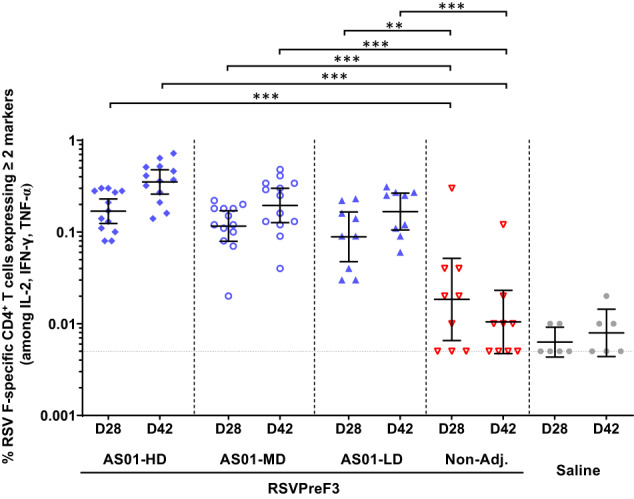
RSV F-specific CD4^+^ T cell responses in mice. Naive CB6F1 mice received three injections, two weeks apart (Days 0, 14, 28). They were administered AS01-adjuvanted RSVPreF3 vaccine formulated with high-dose (HD), medium-dose, (MD) or low-dose (LD) AS01 (*n* = 13, 13 or 9/timepoint, respectively), non-adjuvanted RSVPreF3 vaccine (Non-Adj.; *n* = 9/timepoint), or saline only (controls; *n* = 6/timepoint). Intracellular cytokine staining was performed on splenocytes collected 14 days after the second and third doses (Days 28 and 42, respectively), after restimulation with peptide pools covering the RSVPreF3 sequence. Frequencies of RSV F-specific CD4^+^ T cells expressing at least two markers among IL-2, IFN-γ and TNF-α are presented as geometric means with 95% confidence intervals (bars) and as individual frequencies (symbols). **P < 0.01. ***P < 0.001 (ANOVA for repeated measures). For geometric mean calculations, values of 0% were assigned a value of 0.005% (i.e., half of the minimum detected value), as indicated by the horizontal dotted line.

After the second dose, robust responses of polyfunctional ( ≥ 2 markers-expressing) CD4^+^ T cells were only detected in the three AS01 arms, in which they further increased after the third dose (geometric mean frequencies [GMFs] across these groups: 0.09–0.17% at D28; 0.17–0.35% at D42; Fig. 2). At both timepoints, responses were significantly higher (*P* < 0.01 or *P* < 0.001 depending on the timepoint; ANOVA for repeated measures) in the AS01 arms than in the non-adjuvanted arm (geometric mean ratios [GMRs] AS01/non-adjuvanted: 4.8–9.2 at D28 and 16.0–33.6 at D42). A slight trend towards an AS01 dose-response was also observed at these timepoints.

Response patterns of CD8^+^ T cells expressing at least two markers were similar to those seen in the CD4^+^ T cells, with an increase post-dose 3 and significantly higher (*P* < 0.001; ANOVA for repeated measures) responses for the adjuvanted formulations *vs* the non-adjuvanted vaccine (Supplementary Fig. 3).

Overall, the AS01-based formulations promoted higher polyfunctional T cell immunity as compared to the non-adjuvanted formulation, aligned with the observations made for the humoral responses.

### B cell epitopes of antigenic sites I-V are well conserved between bRSV and RSVPreF3

We next examined whether the robust adjuvanticity of AS01 seen in mice would also be observed in an infection-primed setting, using a model with a higher RSV permissivity as compared to mice^37^. To that aim, we further assessed the impact of AS01 on RSVPreF3 immunogenicity in bRSV infection-primed cows. At prevaccination, mean anti-bRSV IgG levels were comparable across the groups (0.51 or 0.52 EU/mL; Supplementary Table 1). Considering the high-level sequence identity between bRSV and hRSV F proteins^37^ (Supplementary Fig. 4), the hRSV A-based antigen was expected to boost responses cross-reacting with bRSV-specific immune memory. To assess the extent to which the model could faithfully reproduce the human response to RSVPreF3, we first determined the level of B cell epitope conservation across the antigen and the bRSV inactive precursor F (F0).

B cell epitope sequences of the major antigenic sites Ø and I–V of RSV PreF were mapped across the bRSV F0 and RSVPreF3 alignments. Consistent with reported data^18,37,39^, the percentage identity was high for sites I, II, III, IV, and V (91%, 90%, 98%, 100% and 94%, respectively), with sites III and IV being the most conserved. Potencies of NAbs targeting these sites range from low (site I), medium (site II, the target of the therapeutic NAb palivizumab, and site IV) to high (sites III and V)^40^. However, the percentage identity for site Ø, the most neutralization-sensitive and most variable site^18,39^, was only 50%, aligned with the data shown in Supplementary Fig. 4.

### First dose of each RSVPreF3-based vaccine formulation strongly boosts pre-existing neutralization titers in bRSV-primed cows

Using the same RSV strains as those evaluated in mice, we compared the ability to boost pre-existing neutralization titers across different RSVPreF3 formulations in the primed cows (*n* = 8/vaccine group). RSVPreF3 vaccines were either adjuvanted with AS01 or Alum, or non-adjuvanted. Placebo (saline)-treated animals (*n* = 4) were used as controls. All cows received two injections 4 weeks apart (D0, D28), and sera for humoral and CMI response evaluations were collected before (D-7) and 2 and 4 weeks after each dose (D14 and D28, respectively).

**Fig. 3:**
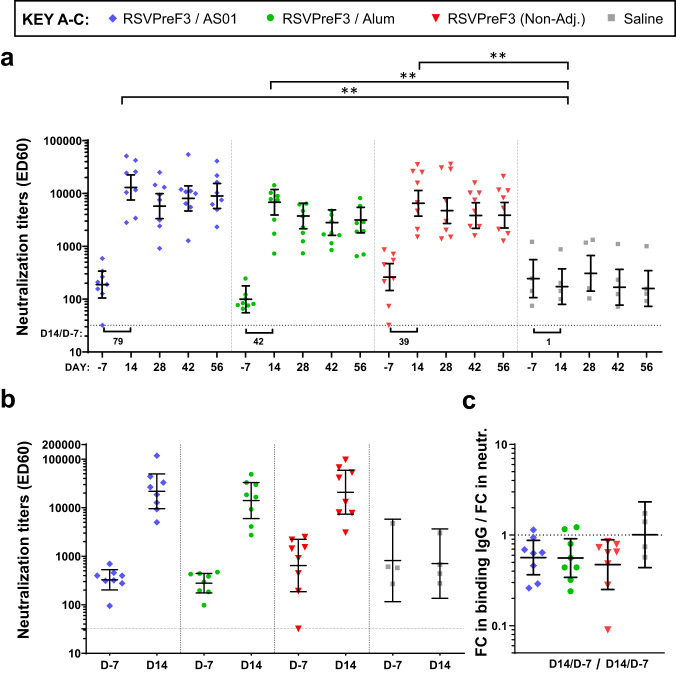
Antibody responses in bovine RSV-primed cattle. Bovine RSV (bRSV) infection-primed cows were injected twice, at Day (D)0 and D28, with the human RSV (hRSV)-based RSVPreF3 antigen formulated with AS01, Alum or without adjuvant (Non-Adj.; *n* = 8/group), or with saline only (controls; *n* = 4). Serum antibody titers were measured before (D-7) and 14 and 28 days after the first and second dose (D14, D28, D42, and D56), and are presented as geometric means with 95% confidence intervals (bars) and as individual values (symbols). ED60, reciprocal of the serum dilution neutralizing 60% of virus. Dotted horizontal lines indicate the limit of detection. **a** hRSV-A neutralization titers against RSV-A Long strain are presented, with geometric means of the fold-changes (FCs) in titers at D14 over D-7 indicated by the values below the horizontal bars. **P < 0.01 (ANCOVA for repeated measures). **b** hRSV-B neutralization titers against the hRSV-B strain 18537 were evaluated. **c** Geometric mean ratios of the D14/D-7 FCs in RSVPreF3-binding immunoglobulin (Ig)G titers, over the D14/D-7 FCs in hRSV-A neutralization titers (neutr.), are presented.

At baseline (D-7), all animals except one each in the AS01 and non-adjuvanted vaccine arms displayed detectable hRSV-A neutralization titers (Fig. 3a). Across the three vaccine arms, baseline titers were boosted by the first dose to levels that were significantly higher (*P* < 0.01; ANCOVA for repeated measures) than for the controls. No statistically significant differences between the vaccine groups were detected at D14. However, both the titers at D14 as well as the fold-increases in these titers from baseline tended to be higher in the AS01 group as compared to the Alum and non-adjuvanted groups (D14 neutralization GMTs [95% CI]: 12563 [7286–21660], 6598 [3791–11484], and 6292 [3608–10974] ED60; GMRs D14/D-7 fold-increases [95% CI]: 79 [42–150], 42 [21–86] and 39 [20–78], respectively). Post-dose two (D42), no boosts compared to the post-dose 1 responses were seen in any vaccine arm, and titers seemed to remain higher for AS01 *vs* Alum but not *vs* the non-adjuvanted group (GMRs [95% CI]: 2.9 [1.3–6.3] and 2.1 [1.0–4.6], respectively). For hRSV-B, boosts of baseline neutralization titers were comparable across the vaccine groups at D14 (the last timepoint evaluated; Fig. 3b).

We next evaluated the impact of a single immunization on the proportion of RSV-A NAbs among RSVPreF3-binding IgG antibodies (Fig. 3c). In the vaccine groups, ratios of the rise in binding titers over the rise in neutralization titers were <1 and similar across the three arms (GMRs D14/D-7 fold-increases: 0.6 for AS01 and Alum; 0.5 for non-adjuvanted), suggesting that the RSVPreF3 antigen favors the induction of functional antibodies.

Thus, both the non-adjuvanted and adjuvanted vaccines were able to boost pre-existing functional NAb responses, with a trend for the AS01-adjuvanted formulation to elicit higher titers. Overall, the data were consistent with those seen previously for the DS-Cav1 antigen in seropositive or seronegative cattle^20,36^.

### Adjuvanted RSVPreF3-based vaccine formulations boost RSV F-specific CD4^+^ T cell responses in bRSV-primed cows

**Fig. 4:**
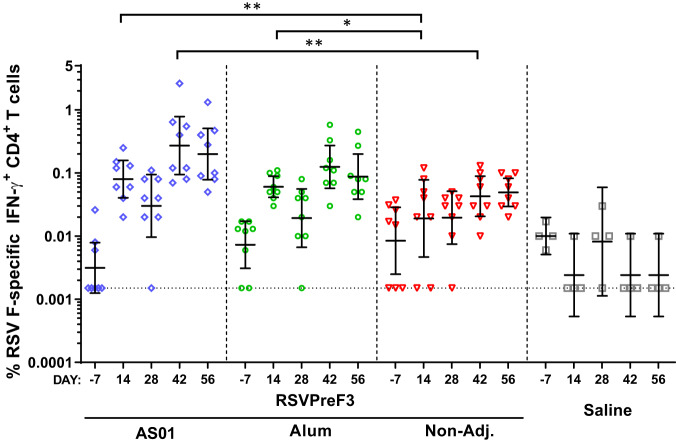
RSV F-specific CD4^+^ T cell responses in bovine RSV-primed cows. Bovine RSV (bRSV) infection-primed cows were injected twice, at Days 0 and 28, with the human RSV-based RSVPreF3 antigen formulated with AS01, Alum or without an adjuvant (Non-Adj.; *n* = 8/group), or with saline only (controls; *n* = 4). Blood samples were collected before (Day-7) and 14 and 28 days after the first and second immunization (Days 14, 28, 42, and 56). Frequencies of RSV F-specific IFN-γ–expressing CD4^+^ T cells were measured by intracellular cytokine staining and flow cytometry of PBMCs after re-stimulation with peptide pools covering the RSVPreF3 sequence. Data are presented as geometric means with 95% confidence intervals (bars) and individual frequencies (symbols). For geometric mean calculations, values of 0% were assigned a value of 0.0015% (i.e., half of the minimum detected value), as indicated by the horizontal dotted line. *P < 0.05. **P < 0.01 (ANCOVA for repeated measures).

ICS analysis of hRSV F-specific IFN-γ–secreting CD4^+^ T cells revealed negligible responses at baseline, which remained low (GMFs ≤0.05%) across subsequent timepoints in the non-adjuvanted vaccine group (Supplementary Fig. 2 [gating strategy] and Fig. 4). By contrast, baseline responses in the AS01 and Alum arms were boosted by the first dose to levels significantly exceeding the D14 levels in the non-adjuvanted arm (GMR [95% CI] over non-adjuvanted: 6.2 [1.7–21.9] with *P* < 0.01 for AS01, and 4.0 [1.2–13.8] with *P* < 0.05 for Alum; ANCOVA for repeated measures).

After the second dose (D42), responses in the vaccine arms tended to increase compared to the post-dose 1 responses. This was most obvious in the AS01 and Alum arms, in which responses remained higher than in the non-adjuvanted arm (GMFs: 0.31% and 0.12% *vs* 0.04%, respectively), though this difference was only significant (*P* < 0.01; ANCOVA for repeated measures) for AS01 (GMRs [95% CI] over non-adjuvanted: 7.5 [2.1–26.6] for AS01; 2.9 [0.9–10.2] for Alum). GMF levels in the two adjuvant groups were not statistically different, but tended to be higher for AS01 at both D14 and D42 (GMRs AS01/Alum [95% CI]: 1.6 [0.4–5.5] and 2.6 [0.7–9.0], respectively). No clear CD8^+^ T cell responses were detected (Supplementary Fig. 5).

Thus, in bRSV pre-exposed cattle the RSVPreF3 vaccines were able to boost RSV F-specific CD4^+^ T cell responses, which were enhanced by an adjuvant (particularly AS01), and, in contrast to the humoral responses, also by a second immunization.

## Discussion

Challenges faced in the development of RSV vaccines for OA include age-related impairments in RSV-specific and naive T cell responses, and heterogeneous levels of baseline immunity, which both complicate the boosting/induction of T cell and/or humoral responses in this population^4,8,10^. Subunit RSV F-based antigens engineered to maintain a stable PreF conformation may possibly benefit from formulation with an adjuvant in order to improve the immune response in OA. As observed with the licensed HZ vaccine^33^, AS01 is capable of robustly boosting antigen-specific immunity in this population. Here, we examined the benefit of AS01 for use in RSVPreF3 vaccine formulations in nonclinical models (naive mice and bRSV infection-primed cattle). We found that the vaccine induced high levels of site Ø-specific IgG responses, confirming that RSVPreF3 was stable in its PreF conformation. In both models, RSVPreF3 vaccines induced RSV-A/B-specific neutralization titers able to neutralize both RSV-A and -B strains. AS01 potentiated both the RSV-A/B-specific neutralizing responses—in terms of titers (mice) or of pre-/post-vaccination fold-increases in titers (bovines)—and the Th1 cytokine-secreting RSV F-specific CD4^+^ T cell responses as compared to responses to non-adjuvanted vaccine. While the effect of AS01 was variable across read-outs and animal models, overall this supported the notion that combining the RSVPreF3 antigen with AS01 improved the antibody and CD4^+^ T cell responses to vaccination, and could hence result in a better vaccine immunogenicity in immunologically impaired populations such as OA.

The AS01-mediated enhancement of antigen-specific antibody and Th1 cytokine-secreting CD4^+^ T cell responses was aligned with observations with other AS01-adjuvanted vaccines in diverse animal models and human populations^25,26,31^. This includes the licensed HZ vaccine, which elicited robust T cell and antibody responses in OA, irrespective of the participants’ age at vaccination^33^. Moreover, the AS01-mediated increase in RSV F-specific CD4^+^ T cell frequencies seen here in both models is consistent with recent data for AS01-adjuvanted RSVPreF3 vaccines when evaluated in OA in the Phase I/II trial^21^.

Adjuvanticity of AS01 relies on the synergistic expression of innate IFN-γ by both QS-21 and the TLR4 agonist MPL^29,41^. By activating mostly IFN-driven pathways, AS01 increases cytokine-secreting CD4^+^ T cell responses that in turn support development of other immune cells, such as T follicular helper (T_FH_) cells. The latter cells, which were detected in human blood following administration of AS01-containing vaccines^42,43^, can improve antibody functionality by providing help to B cells. Here, in the mice, AS01 enhanced secretion of site Ø epitope-binding antibodies—known to have a higher neutralizing potency than palivizumab^16,18^—which hinted at a stronger functional antibody response in these animals. In contrast, MPL alone combined with DS-Cav1 also increased murine binding antibody titers, but not site Ø-specific titers^36^. Our data are thus consistent with a model of improved T_FH_ responses, mediated by an MPL/QS-21 synergy^29,41^. This underscores the relevance of selecting an adjuvant able to promote/boost CD4^+^ T cell responses—and thus likely also T_FH_ responses—for use in vaccines for OA. Accordingly, clinical trial data for other vaccines, evaluated in young adults, suggested that AS01 increased antibody avidity^27^. Both IFN-γ production (which has been linked to RSV clearance^8,14,15^) and T_FH_-cell differentiation/abundance decline with age^11,44^. It is therefore promising that in the Phase I/II trial, the presence of the adjuvant resulted in significantly higher frequencies of IFNγ-producing RSV F-specific CD4^+^ T cells in the OA recipients of the AS01-adjuvanted RSVPreF3 vaccine^21^. Moreover, as seen for other AS01-adjuvanted vaccines^27,45^, the RSVPreF3 vaccine stimulated activation of several Fc-mediated effector functions, such as antibody‐dependent NK-cell activation^21^ which may play a role in protection against RSV^46^.

Interestingly, the bovine data suggest that the memory B cell pool in the cows would have matched the vaccine antigen near-completely for sites I–V, but to a much lesser extent for site Ø given the low RSVPreF3/bRSV homology of this site. Nonetheless, as neutralizing activity strongly relies on the abundance of site Ø-specific antibodies^16^, the detection of high neutralization titers (~12,500 ED60) with RSVPreF3/AS01 in bovine sera is encouraging. This data suggests that the antigen, irrespective of the formulation, was capable of selecting and/or activating site Ø-specific memory B cells, even though these cells would have had to compete with the site I–V-specific memory B cells.

The lack of statistical difference between AS01 and Alum for RSV F-specific CD4^+^ T cell responses in the cows contrasted with the lower adjuvanticity of Alum compared to AS01 in innate or adaptive responses seen against different antigens in mice, monkeys, and humans^27,31,41,47–49^. Besides the small sample size in this model (*n* = 8/vaccine group), other factors may also explain this result. While some effect for QS-21 was expected in the cows based on published data for saponins (a common veterinary adjuvant also used in cattle^50,51^), MPL is only weakly immunogenic in bovines due to the different expression of bovine TLR4^52,53^. This may also have decreased any synergistic effects between these AS01 components^29,41^ in the bovine model, further deepening the divergence between the two evaluated species. Another factor in the inter-species difference in adjuvanticity may have been the extensive level of bacterial lipopolysaccharide and RSV pre-exposure of the cows *vs* the immune status of the laboratory mice. Finally, as our study was limited by the lack of measurements of bovine multifunctional T cells, deeper analyses of secreted cytokines beyond IFN-γ, or of gene expression profiles, may allow detecting clearer differences between Alum and AS01 in this model. Thus, the adjuvanticity of AS01, when combined with RSVPreF3, depended on both the host species and the RSV priming levels, as also noted for DS-Cav1^36^ and (with respect to priming) aligned with the RSVPreF3 data in OA^21^.

Species-specific effects were also observed for the CD8^+^ T cell responses. The latter were detected only in the mice. After vaccination with protein antigens, even when formulated with AS01, such responses are typically not observed, or detected at low levels, in human blood^25,33,54^, as corroborated by the recent RSVPreF3/AS01 data^21^. Due to diverging HLA expression and antigen presentation, the murine CD8^+^ T cell responses were not surprising and also seen for other proteins^29,55^, though this effect may be antigen-dependent given the inconsistent responses seen for VZV gE in mice^41,49^. Differences with VZV gE were also noted for the AS01 dose-response in mice, which was not seen here with RSVPreF3 but was detected in gE-specific responses in VZV–pre-immunized mice^41,49^. This highlights that besides the host and the population priming levels, also the antigen controls the adjuvanticity (and dose-effects) of AS01, as noted for other antigen/adjuvant combinations^35,36^. The latter was confirmed by the current data showing significantly higher RSV-A-specific neutralization titers for RSVPreF3/AS01 as compared to AS01-adjuvanted RSV PostF vaccine. Apart from their different propensity to develop CD8^+^ T cell responses, the overall T cell biology is mostly analogous between humans and mice, with both species favoring antibody and/or CD4^+^ T cell induction in response to protein antigens. This suggests that specific aspects of AS01 adjuvanticity seen elsewhere in mice, such as the innate cytokine profiles and efficient T cell priming in the lymph node^25,28^, can be reproducible in humans (though less so in cattle).

In conclusion, our preclinical data showed that a formulation of the PreF-stabilized RSVPreF3 antigen with AS01 induced/boosted potent polyfunctional RSV F-specific CD4^+^ T cell responses, as well as RSV-A/B-specific neutralizing responses which were in great part directed toward the neutralization-sensitive antigenic site Ø. Collectively, these results supported the evaluation of AS01-adjuvanted formulations during the clinical development of the RSVPreF3 candidate vaccine against RSV-associated LRTD in OA.

Following the Phase I/II study^21^, Phase IIb and III trials of the AS01_E_-adjuvanted RSVPreF3 vaccine in OA have been initiated (NCT04657198, NCT04732871, NCT04841577, NCT05059301, NCT04886596). These trials are currently in progress or have been completed. Recent data from the pre-specified efficacy analysis of the Phase III (AReSVi-006) trial demonstrated that the vaccine candidate met the primary endpoint of efficacy against RSV LRTD, conferring a statistically significant and clinically meaningful efficacy in the OA study population^56^. The RSVPreF3/AS01 vaccine has recently been approved by the European Commission, based on the European Medicines Agency’s recommendation, and by the United States Food and Drug Administration, for the prevention of LRTD caused by RSV in individuals 60 years of age and older.

Finally, in the bRSV-primed cow model, neither the presence of AS01 nor the number of immunizations appeared to impact the RSVPreF3-induced RSV-neutralization titers, while both of these factors were shown to enhance the RSV F-specific CD4^+^ T cell responses. These results mirror the observations made in OA following RSVPreF3/AS01 administration^21^, and support the relevance of the bRSV-primed adult cow model for the evaluation of RSV candidate vaccines for OA.

## Methods

### Ethics statement

In vivo bovine experiments were conducted at the Centre d’Economie Rural (CER) Groupe facilities (Marloie, Belgium). Murine experiments were performed in GSK’s AAALAC-accredited animal facilities (Rixensart, Belgium). Husbandry/experiments were ethically reviewed and performed in accordance with Belgian and European laws/guidelines/policies for animal experimentation, housing, and care (Treaty ETS #123, Belgian Royal Decree 29-05-2013; European Directive 2010/63/EU), and GSK’s Policy on the Care, Welfare, and Treatment of Animals. Protocols were approved by the local ethical review committees of GSK (mice: #P004/26/01; cows: #P00/000/00/Av1.00) and CER (cows; #CE/Santé/ET/012).

### Vaccines

RSVPreF3 is a PreF-stabilized antigen based on the F amino acid sequence from the prototype RSV A2 strain, and is expressed in Chinese hamster ovary (CHO) cells^21^. Good Manufacturing Practice (GMP) and non-GMP antigen lots were used: non-GMP lots for the mice, and a GMP Phase I lot for the bovines. The RSV PostF antigen used in a separate experiment in mice was expressed in CHO cells. RSV PostF protein is not stabilized to maintain the PreF conformation and therefore naturally adopts the PostF state^57^. Mice received 0.5 μg RSVPreF3/injection or, in the separate experiment, 2 μg of either RSVPreF3 or RSV PostF per injection. A human dose of AS01_B_ (0.5 mL) contains 50 µg MPL (3-*O*-desacyl-4′-monophosphoryl lipid A; produced by GSK) and 50 µg QS-21 (*Quillaja saponaria* Molina, fraction 21; licensed by GSK from Antigenics LLC, a wholly owned subsidiary of Agenus Inc., a Delaware, USA corporation) in a liposomal formulation. Just prior to immunization, the lyophilized antigen was reconstituted with either phosphate-buffered saline (PBS; 0.9% NaCl) or one of three different AS01 doses. High-dose (HD), medium-dose (MD) and low-dose (LD) AS01 corresponded to 1/10th, 1/20th, and 1/40th of the human dose of AS01_B_, amounting per injection to 5, 2.5, or 1.25 µg, respectively, of each of the immunostimulants (MPL, QS-21) in a liposomal formulation. Alum-adjuvanted vaccines used in a separate experiment in mice contained 50 μg Al(OH)_3_/injection. Immunized cattle received 420 µg RSVPreF3/injection, representing the weight-proportional dose of cattle vs human of 7× the middle dose (among 30, 60, and 120 µg doses) evaluated in the Phase I/II study in OA^21^. AS01 recipient bovines received 7× the human dose of AS01_B_, i.e., 3.5 mL containing 350 µg MPL and 350 µg QS-21/injection. Alum recipient bovines received 3500 µg Al(OH)_3_/injection.

### Animals and immunizations

Hundred female CB6F1 mice (6–8 weeks old; Envigo, Horst, The Netherlands) were housed under normal conditions with unlimited access to food and filtered tap water. They were anesthetized with isoflurane gas for their electronic identification. They were randomly segregated into five groups to receive three 50 μL intramuscular injections, two weeks apart, at Day (D)0 (priming dose), D14 and D28. Animals were administered RSVPreF3 vaccines formulated either with AS01 (HD: *n* = 26; MD: *n* = 26; LD: *n* = 18) or without adjuvant (*n* = 18), and a control group received saline only (*n* = 12). Two weeks after the second and third dose (D28 and D42, respectively), half of the mice/group were first bled to collect sera for antibody measurements, then euthanized by an intraperitoneal injection of a pentobarbital overdose to harvest spleens for T cell evaluations. As part of the terminal bleeding, they were anesthetized by injection of a mix of atropine, ketamine, droperidol, and fentanyl citrate. In a similarly designed experiment, a total of 70 mice were injected with either RSVPreF3 or RSV PostF vaccines (each formulated with AS01 [HD], Alum or without adjuvant) or saline only (*n* = 10/group).

Twenty-eight 3–9-year-old cows (*Bos taurus*) purchased from local farmers were confirmed to have not received any prior vaccinations against bRSV and to be seropositive for anti-bRSV IgG by semi-quantitative Enzyme-Linked Immuno-Sorbent Assay (ELISA) before treatment. Animals were group-housed, had access to hay and drinking water ad libitum, and received 2 kg pellets daily. They were randomized based on their baseline anti-bRSV IgG titers into three vaccine groups (*n* = 8/group) and a saline control group (*n* = 4) to receive two 3.5-mL intramuscular injections, 4 weeks apart (D0, D28) in the neck. Immunized animals received RSVPreF3 formulated with Alum or AS01, or non-adjuvanted vaccine. Sera were collected before immunization (D-7), and 14 or 28 days after either the first dose (D14, D28), or the second dose (D42, D56) without using anesthesia. Daily monitoring by trained staff demonstrated that clinical health statuses and injection-site reactions were acceptable throughout the study. At the end of the study, cows were euthanized via captive bold shot and then exsanguinated.

### RSV neutralization assays

Sera were serially diluted two-fold in RSV medium (Biorich DMEM 3% FBS, 2 mM L-glutamine, 50 µg/mL gentamicin). Sample and positive human serum (BEI Resources NR-4020; NIBSC 16/284) dilutions were mixed with hRSV A Long (American Type Culture Collection [ATCC], VR-26) or hRSV B 18537 (ATCC, VR-1580) diluted to approximately 100 plaque-forming units/well, and incubated for 2 h at 35 °C. After incubation, the virus-serum mixture was transferred to Vero cell-seeded plates (15000 cells/well), with virus-only wells as 100% infectivity control. Plates were incubated for 2 h at 35 °C, then the medium was removed and RSV medium containing 0.5% carboxymethylcellulose (Sigma C4888) was added to all wells. The plates were incubated for 42 h at 35 °C. Before staining, the plates were washed with PBS and fixed overnight at 4 °C with paraformaldehyde (at 1%). Staining was performed with goat anti-RSV antibody (Biodesign B65860G), followed by rabbit anti-goat IgG-horse radish peroxidase (Millipore AP106P/Rockland 605-403-B9). After the antibody staining, True Blue substrate (KPL 71-00-68) was added to all wells to reveal the infectious foci. Plates were scanned using a ScanLab/Axiovison reader. Reciprocal NAb titers were expressed in effective dilution (ED)60, determined as the reciprocal of the serum dilution causing 60% reduction in the number of plaques as compared to the control wells (virus only, no serum).

### RSVPreF3-binding IgG ELISA

Anti-RSVPreF3 IgG antibodies in bovines were quantified by ELISA using RSVPreF3 antigens as coating. Antigens were diluted at a final concentration of 2 μg/mL in PBS, and adsorbed overnight at 4 °C using 96-well microtiter plates (Maxisorp Immuno-plate, Nunc, Denmark). Plates were then incubated (1 h, 37 °C) with PBS + 0.1% Tween20 + 1% bovine serum albumin (BSA; saturation buffer). Sera diluted in saturation buffer were added to the RSVPreF3-coated plates and incubated (1 h, 37 °C). Plates were washed four times with PBS 0.1% Tween20, and peroxidase-conjugated goat anti-bovine IgG (H + L) (Invitrogen #A18751) diluted 1:2000 in saturation buffer was added to each well and incubated (30 min, 37 °C). Plates were washed as indicated above and rinsed with deionized water before being incubated (15 min, room temperature [RT]) with 3,3’,5,5’-tetramethylbenzidine diluted ¾ in 0.1 M citrate buffer pH 5.8. The reaction was stopped with 2 N H_2_SO_4_ and plates were read at 450/620 nm using a microplate reader (Versamax OD reader, Molecular Devices). Titers were calculated from a reference by SoftMaxPro (using a four-parameter equation) and expressed in ELISA Units (EU)/mL.

### Antibody competition ELISA

A competition ELISA was performed to determine the amounts of antibodies targeting the PreF-exclusive antigenic site Ø in murine sera. The D25 antibody (tracer) was biotin-conjugated using a commercial kit according to manufacturer instructions (EZ Link NHS-PEG4 Biotin, No-Weigh Format, Thermo Scientific). ELISA plates (96-well, Immuno F96 MaxiSorp, Nunc) were coated with 100 μL/well of RSVPreF3 diluted to 2 μg/mL in PBS. Following overnight incubation at 4 °C, wells were washed with PBS containing 0.05% (w/v) Tween20 (wash buffer) prior to blocking with 1% (w/v) BSA in PBS for 90 min at RT. Mouse serum samples (starting 1:10) or unlabeled D25 monoclonal antibody (standard, starting at 5 μg/mL) were serially diluted two-fold in PBS containing 1% (w/v) BSA and 0.1% (w/v) Triton X-100 (sample buffer). Sample and standard dilutions were combined in equal volumes with 8 ng/mL tracer. The ELISA plates were washed and tracer-sample/standard mixtures were transferred to the plates. Eight wells contained tracer only for determination of the tracer signal (tracer-only binding). Plates were then washed and incubated with horse radish peroxidase-conjugated avidin (Vector cat# A-2004) diluted 1:10,000 in sample buffer at 100 μL/well for 1 h at RT, followed by a wash and incubation for 20 min with 100 μL/well of TMB substrate (Bio-Rad 172-1072) at RT. Following incubation, the reaction was stopped by adding 100 μL/well of 2.0 N sulfuric acid. The optical density was determined at 450/620 nm using a microplate reader (Versamax OD reader, Molecular Devices). Percent inhibition of the tracer-only binding was calculated for each standard or sample dilution and plotted according to concentration (standards) or dilution (samples). For standards, the concentration of unlabeled D25 leading to 50% inhibition of the corresponding tracer (EC50) was calculated in SoftMAxPro GxP v 5.3. For samples, the dilution corresponding to 50% inhibition was calculated in a similar manner.

### ICS

Murine splenocytes and bovine peripheral blood mononuclear cells (PBMCs) were isolated from homogenized spleens and heparinized blood, respectively. For both models, cells were plated at 10^6^ cells/well and stimulated with a pool of 15-mer peptides (1 μg/mL) overlapping by 11 amino acids covering the RSVPreF3 sequence, in the presence of anti-CD28 (clone 37.51 for mice; clone L293 for cows) and anti-CD49d (clone 9C10/MFR4.B for mice; clone L25 for cows) antibodies, or were left unstimulated (controls). Phorbol 12-myristate 13-acetate (PMA) ionomycine was used as positive control for the in vitro T cell activation and stimulation of cytokine production. After 2 h at 37 °C, Brefeldin A was added for another 4 h. Plates were left overnight at 4 °C. Cells were centrifuged and resuspended in Flow Buffer (PBS 1×, 1% FCS). Murine cells were incubated at 4 °C, first for 10 min with anti-CD16/32 antibody, then for 30 min with anti-CD4-V450 and anti-CD8-PerCp-Cy5.5 antibodies and Live/Dead-PO (Invitrogen). Bovine PBMCs were incubated with Live/Dead Near-IR (Invitrogen; 30 min, RT), washed, stained (Alexa Fluor 647-conjugated mouse anti-bovine CD4; FITC-conjugated mouse anti-bovine CD8) for 30 min, centrifuged and washed. Splenocytes/PBMCs were fixed/permeabilized in 200 μL Cytofix-Cytoperm, incubated (20 min, 4 °C), washed in 1×Perm/Wash buffer, and stained for 1 h (mice) or 2 h (cattle) at 4 °C in 1×Perm/Wash buffer with antibodies (mice: anti-IL2-FITC, anti-IFNγ-APC and anti-TNFα-PE; cattle: PE-conjugated mouse anti-bovine IFN-γ). Cells were then washed twice with 1×Perm/Wash buffer in PBS. Fluorescent events were acquired using LSR2 (mice) or LSRFortessa (cattle) flow cytometers, and analyzed with FlowJo software (Tree Star). Reagents/devices were from BD Biosciences unless specified otherwise.

### RSVPreF3/bRSV conservation analyses

Conformationally restricted antigenic sites of the RSV PreF protein surface (amino acids accessible to antibodies for each reported site) were mapped using published data^39^, crystal structures, and the MOE residue-property tool. Antigenic conservation and structural impacts of identified mutations were evaluated, retaining only the prevalent polymorphisms for each antigenic site position (≥5% of the total RSV-A/B sequences). By searching the NCBI database for “*bovine Respiratory Syncytial virus*”, 421 sequences were mined, checked for RB94 (AM746678) sequences, then aligned using the MUSCLE program to mine F0 protein sequences. Twelve full-length bRSV F0 nucleotide sequences were extracted, translated into 12 protein sequences using the TRANSEQ program, and MUSCLE-aligned. Twelve F0 ectodomain sequences were extracted and aligned with RSVPreF3 using MUSCLE to assess the conservation of B cell epitopes.

### Statistics

Descriptive statistics were calculated using SAS version 9.4 (SAS Institute Inc., Cary, NC, USA) or Prism 6.0 (GraphPad, San Diego, CA, USA). NAb titers below the assay cut-offs were assigned a value equal to the cut-offs, i.e., 5 or 32 ED60 for mice and cows, respectively. For geometric mean calculations of T cell frequencies, values of 0% were assigned a value of 0.005% (CD4^+^/CD8^+^ T cells; mice), 0.0015% (CD4^+^ T cells; cows), or 0.0026% (CD8^+^ T cells; cows), each equaling half the minimum observed value in the respective models. For all comparative analyses except those in Supplementary Fig. 1, ANOVA (mice) or ANCOVA (cows; adjusted for baseline) models for repeated measures (with time/treatment/interactions) were fitted on log_10_-transformed geometric mean titers/frequencies in SAS version 9.4. For the data in Supplementary Fig. 1, a one-way ANOVA with heterogeneous variance (as determined by Levene’s test) was fitted to the data using R (v4.1.1); the NaCl group was excluded due to the absence of variability.

### Reporting summary

Further information on research design is available in the Nature Research Reporting Summary linked to this article.

### Supplementary information

Supplementary Information

Reporting Summary

## Data Availability

The authors declare that data supporting the findings of this study are available within the paper and its Supplementary Information files.
