# Supplementary material for: Immunogenicity of an AS01-adjuvanted respiratory syncytial virus prefusion F (RSVPreF3) vaccine in animal models
Source: NPJ Vaccines. 2023 Sep 29;8:143. doi: 10.1038/s41541-023-00729-4 (PMC10541443; doi:10.1038/s41541-023-00729-4)
Supplement: Supplementary file 1 — Supplementary Information [file 41541_2023_729_MOESM1_ESM.pdf]

Supplementary Information

Immunogenicity of an AS01-adjuvanted respiratory syncytial virus prefusion F (RSVPreF3) candidate vaccine in animal models

Badiaa Bouzya *et al*.

Supplementary Table 1. Individual binding antibody concentrations in bRSV-primed cows

		Anti-bRSV IgG at prevaccination (day -7)		
Animal identification code	Treatment group	Concentration (EU/mL)	Mean	Standard deviation
V17	RSVPreF3 / Alum	0.33	0.51	0.13
V18	RSVPreF3 / Alum	0.60		
V19	RSVPreF3 / Alum	0.49		
V20	RSVPreF3 / Alum	0.58		
V21	RSVPreF3 / Alum	0.39		
V22	RSVPreF3 / Alum	0.56		
V23	RSVPreF3 / Alum	0.41		
V24	RSVPreF3 / Alum	0.72		
V25	RSVPreF3 / AS01	0.37	0.52	0.17
V26	RSVPreF3 / AS01	0.33		
V27	RSVPreF3 / AS01	0.48		
V28	RSVPreF3 / AS01	0.68		
V29	RSVPreF3 / AS01	0.81		
V30	RSVPreF3 / AS01	0.43		
V31	RSVPreF3 / AS01	0.46		
V32	RSVPreF3 / AS01	0.63		
V33	RSVPreF3 / PBS	0.52	0.51	0.18
V34	RSVPreF3 / PBS	0.65		
V35	RSVPreF3 / PBS	0.33		
V36	RSVPreF3 / PBS	0.30		
V37	RSVPreF3 / PBS	0.78		
V38	RSVPreF3 / PBS	0.56		
V39	RSVPreF3 / PBS	0.63		
V40	RSVPreF3 / PBS	0.30		
V41	PBS	0.58	0.51	0.13
V42	PBS	0.58		
V43	PBS	0.32		
V44	PBS	0.56		

IgG, immunoglobulin G. EU, ELISA units. PBS, phosphate-buffered saline.

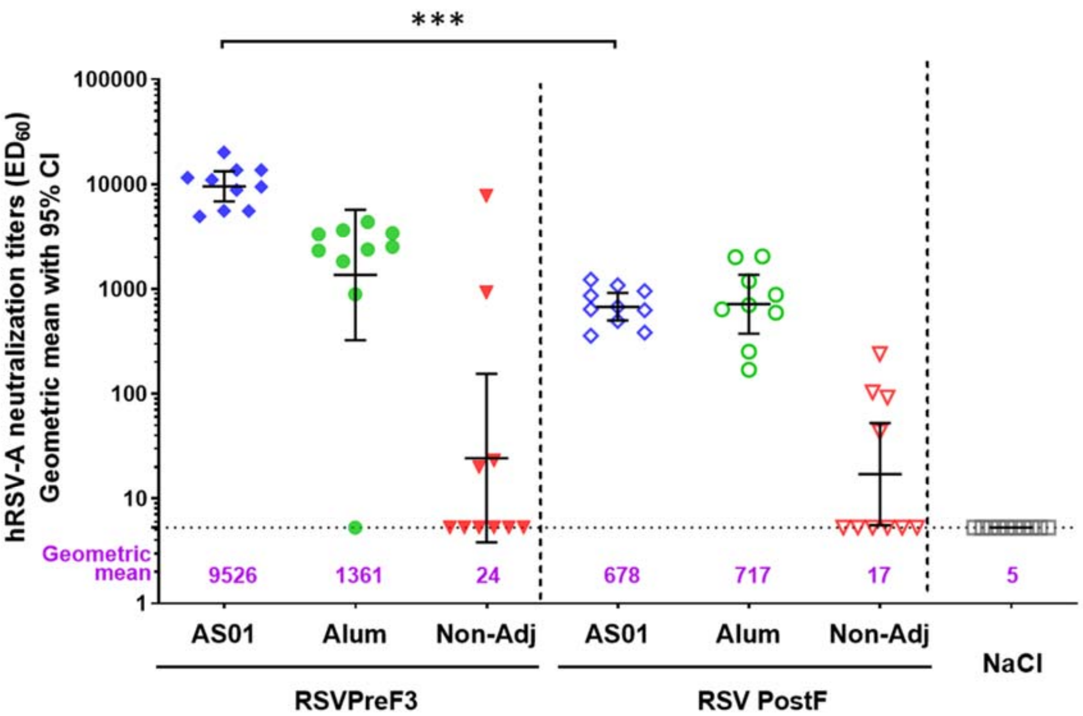

Supplementary Fig. 1. Adjuvanticity of AS01 combined with RSVPreF3 vs an RSV PostF antigen in mice. Naive CB6F1 mice (n=10/group) received three injections, two weeks apart (Days 0, 14, 28), and neutralization titers (obtained with the RSV-A Long strain) were measured 14 days after dose 3. Mice were administered different formulations of either RSVPreF3 or an RSV PostF antigen, or saline only (NaCl; controls). Vaccines were either adjuvanted with AS01 (high dose) or Alum, or non-adjuvanted (Non-Adj). Data are presented as geometric means titers with 95% confidence intervals (CI; bars) and as individual titers (symbols), and expressed as reciprocals of the serum dilution neutralizing 60% of virus (ED60). The horizontal dotted line indicates the limit of detection. ***, *P* <0.001 (one-way ANOVA with heterogeneous variance).

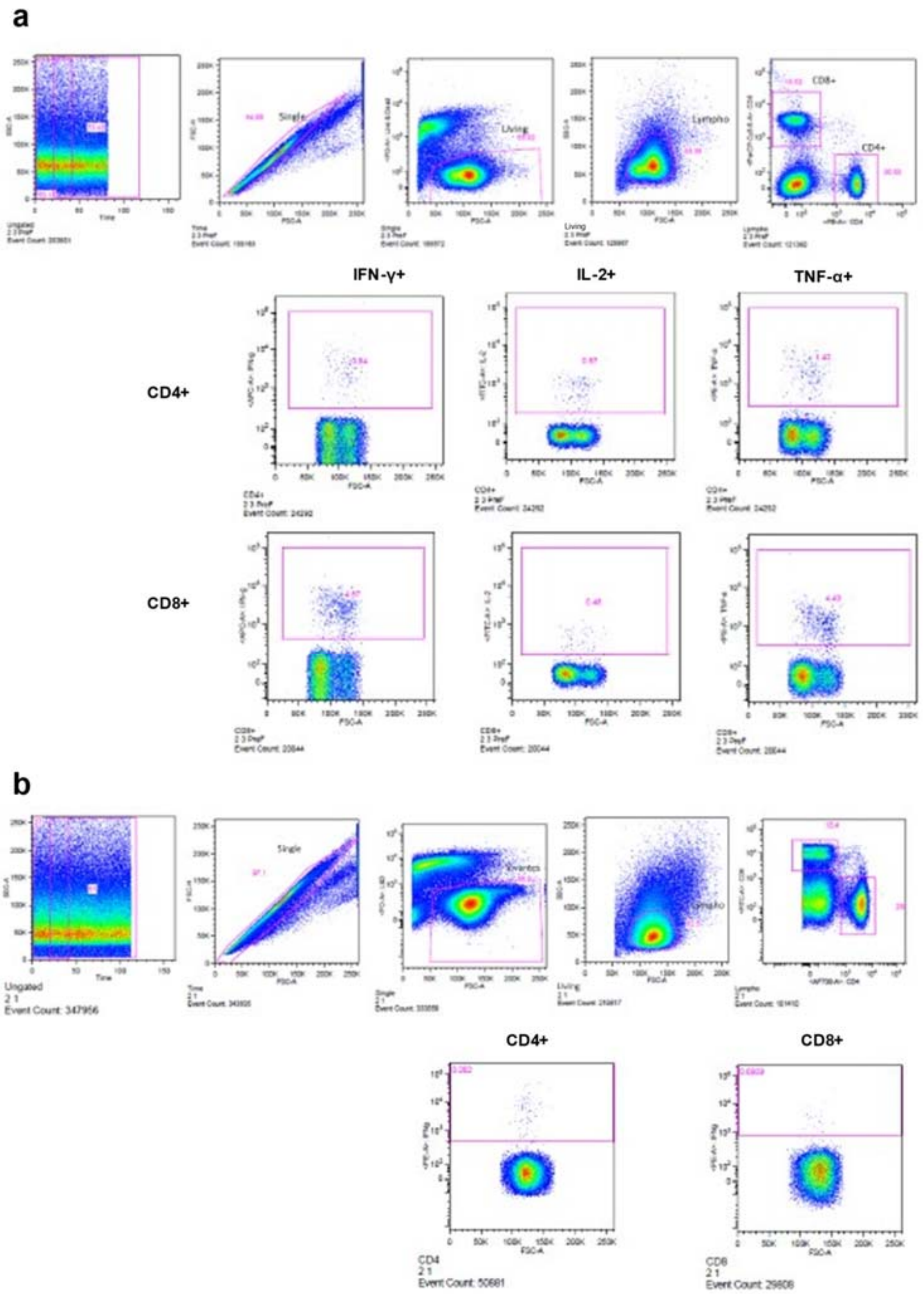

Supplementary Fig. 2. Flow cytometry gating strategy. RSV F-specific cytokine-expressing T cells were assessed using ICS and flow cytometry. Results are representative of the range of responses seen for all samples. Numbers in pink font in the quadrant gates of the plots present each distinct population based on their marker expression. **(a)** Gating used for the assessment of RSV F-specific IL-2-, TNF-α- and/or IFN-γ-expressing $CD4^+$ and $CD8^+$ T cells in murine samples collected 14 days after the third immunization with AS01-adjuvanted RSVPreF3 vaccine. **(b)** Gating used for the assessment of RSV F-specific IFN-γ-expressing $CD4^+$ and $CD8^+$ T cells in bovine samples collected after the second immunization with AS01-adjuvanted RSVPreF3 vaccine.

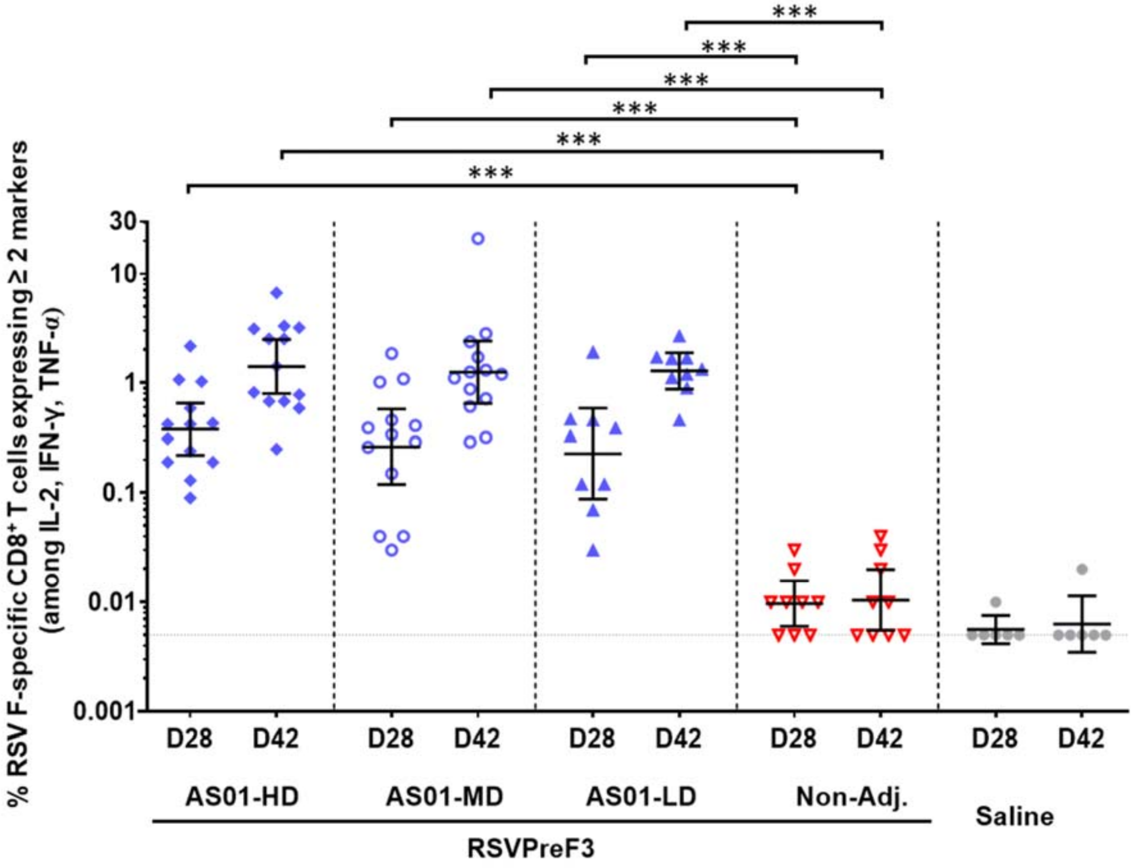

Supplementary Fig. 3. RSV F-specific CD8$^+$ T-cell responses in mice. Naive CB6F1 mice received three injections, two weeks apart (Days 0, 14, 28). They were administered AS01-adjuvanted RSVPreF3 vaccine (formulated with high-dose [HD], medium-dose [MD] or low-dose [LD] AS01; n=13, 13 or 9/timepoint, respectively), non-adjuvanted RSVPreF3 vaccine (n=9/timepoint), or saline only (controls; n=6/timepoint). Intracellular cytokine staining was performed on splenocytes collected 14 days after the second and third doses (Days 28 and 42, respectively), after restimulation with peptide pools covering the RSVPreF3 sequence. Frequencies of RSV F-specific CD8$^+$ T cells expressing at least two markers among IL-2, IFN-γ and TNF-α are presented as geometric means with 95% confidence intervals (bars) and as individual frequencies (symbols). ***, P <0.001 ANOVA for repeated measures. For geometric mean calculations, values of 0% were assigned a value of 0.005% (i.e., half of the minimum detected value), as indicated by the horizontal dotted line.

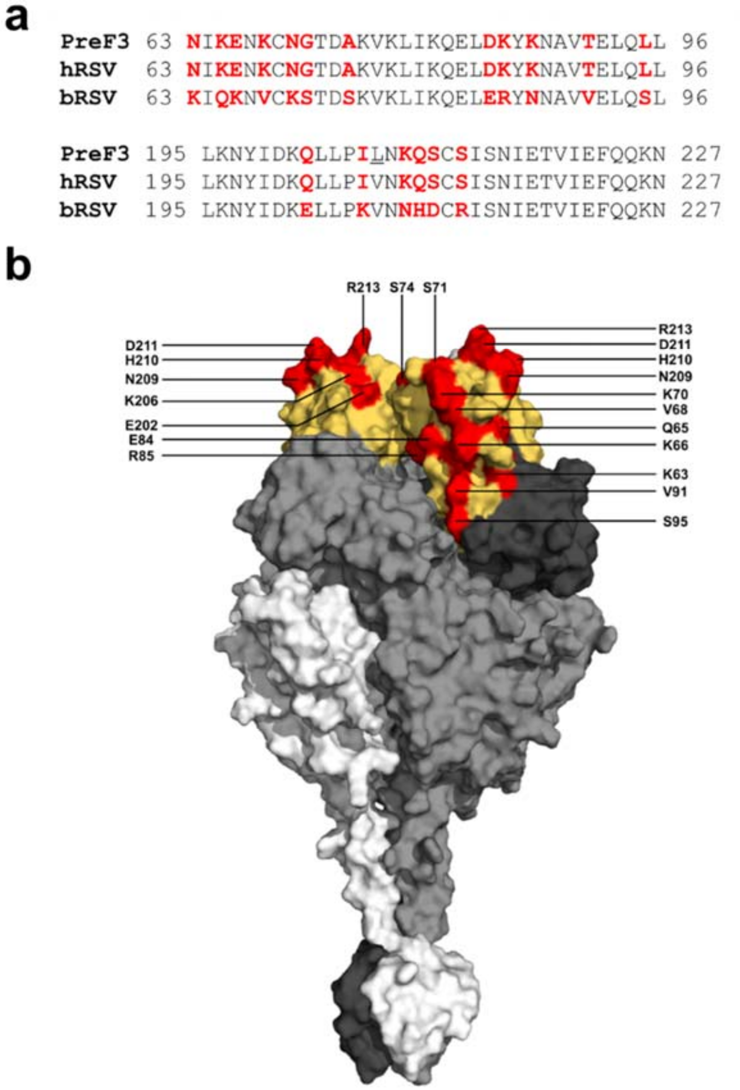

Supplementary Fig. 4. Mapping of the amino acid differences between PreF3, human and bovine RSV F protein antigenic site Ø. (a) Sequence alignments show the antigenic site Ø alignments of the RSVPreF3, human (h)RSV and bovine (b)RSV consensus sequences. Differences between the RSVPreF3, hRSV and bRSV residues are indicated in bold red font. The mutation introduced in RSVPreF3 to stabilize the F protein prefusion conformation is indicated in underlined font. **(b)** The bRSV amino acids found to differ from hRSV were mapped at the surface of a bRSV prefusion conformation structure (PDB ID: 5TDG). Colors indicate the three protomers depicted at the surface (grey shades), the antigenic site Ø in two of the protomers (yellow), and the amino acids found to differ between bRSV and hRSV (red).

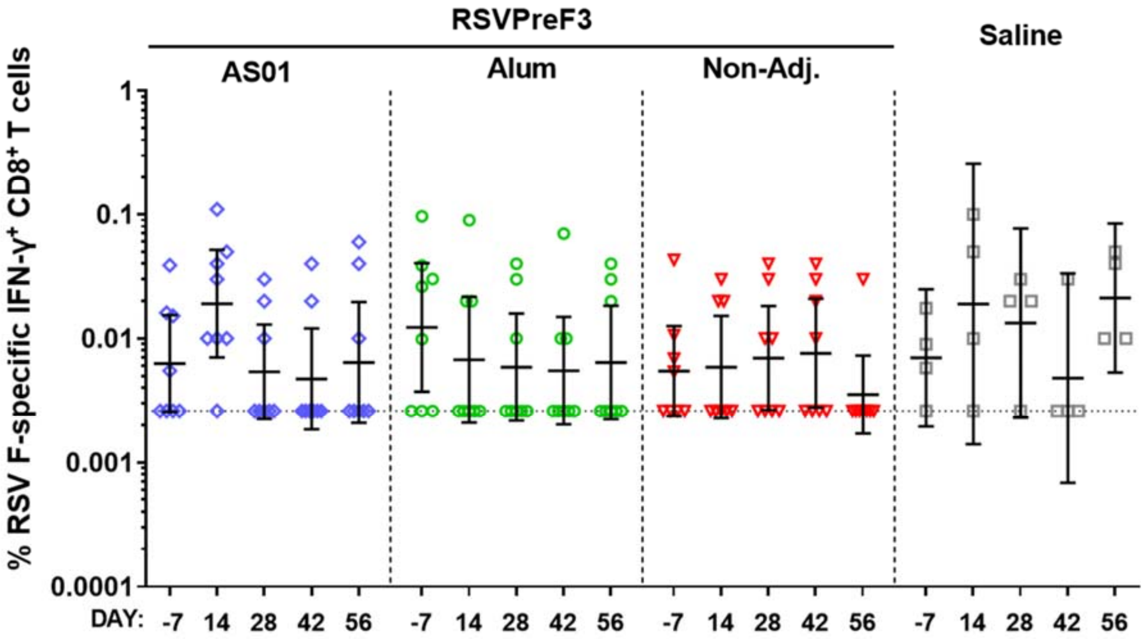

Supplementary Fig. 5. RSV F-specific CD8$^+$ T-cell responses in bovine RSV-primed cows. Bovine RSV (bRSV) infection-primed cows were injected twice, at Days 0 and 28, with the human RSV-based RSVPreF3 antigen formulated with AS01, Alum or without an adjuvant ('Non-Adj'; n=8/group), or with saline only (controls; n=4). Blood samples were collected before (Day -7) and 14 and 28 days after the first and second immunization (Days 14, 28, 42, and 56). Frequencies of RSV F-specific IFN-γ–expressing CD8$^+$ T cells were measured by intracellular cytokine staining and flow cytometry of PBMCs after re-stimulation with peptide pools covering the RSVPreF3 sequence. Data are presented as geometric means with 95% confidence intervals (bars) and individual frequencies (symbols). For geometric mean calculations, values of 0% were assigned a value of 0.0026% (i.e., half of the minimum detected value) as indicated by the horizontal dotted line.
