# Supplementary material for: Immunogenicity of an AS01-adjuvanted respiratory syncytial virus prefusion F (RSVPreF3) vaccine in animal models
Source: NPJ Vaccines. 2023 Sep 29;8:143. doi: 10.1038/s41541-023-00729-4 (PMC10541443; doi:10.1038/s41541-023-00729-4)
Supplement: Supplementary file 2 — Reporting Summary [file 41541_2023_729_MOESM2_ESM.pdf]

nature portfolio

Corresponding author(s): Clarisse Lorin

Last updated by author(s): Jun 13, 2023

Reporting Summary

Nature Portfolio wishes to improve the reproducibility of the work that we publish. This form provides structure for consistency and transparency in reporting. For further information on Nature Portfolio policies, see our Editorial Policies and the Editorial Policy Checklist.

Statistics

For all statistical analyses, confirm that the following items are present in the figure legend, table legend, main text, or Methods section.

n/a	Confirmed	
☐	☒	The exact sample size (n) for each experimental group/condition, given as a discrete number and unit of measurement
☐	☒	A statement on whether measurements were taken from distinct samples or whether the same sample was measured repeatedly
☐	☒	The statistical test(s) used AND whether they are one- or two-sided<br>*Only common tests should be described solely by name; describe more complex techniques in the Methods section.*
☐	☒	A description of all covariates tested
☒	☐	A description of any assumptions or corrections, such as tests of normality and adjustment for multiple comparisons
☐	☒	A full description of the statistical parameters including central tendency (e.g. means) or other basic estimates (e.g. regression coefficient) AND variation (e.g. standard deviation) or associated estimates of uncertainty (e.g. confidence intervals)
☐	☒	For null hypothesis testing, the test statistic (e.g. F, t, r) with confidence intervals, effect sizes, degrees of freedom and P value noted<br>*Give P values as exact values whenever suitable.*
☒	☐	For Bayesian analysis, information on the choice of priors and Markov chain Monte Carlo settings
☒	☐	For hierarchical and complex designs, identification of the appropriate level for tests and full reporting of outcomes
☒	☐	Estimates of effect sizes (e.g. Cohen's d, Pearson's r), indicating how they were calculated

Our web collection on statistics for biologists contains articles on many of the points above.

Software and code

Policy information about availability of computer code

Data collection	LSR2 (mice) or LSRFortessa (cattle) flow cytometers were used for collecting flow cytometry data.
Data analysis	FlowJo software (Tree Star); SAS version 9.4 (SAS Institute Inc.); Prism 6.0 (GraphPad).

For manuscripts utilizing custom algorithms or software that are central to the research but not yet described in published literature, software must be made available to editors and reviewers. We strongly encourage code deposition in a community repository (e.g. GitHub). See the Nature Portfolio guidelines for submitting code & software for further information.

Data

Policy information about availability of data

All manuscripts must include a data availability statement. This statement should provide the following information, where applicable:

- Accession codes, unique identifiers, or web links for publicly available datasets
- A description of any restrictions on data availability
- For clinical datasets or third party data, please ensure that the statement adheres to our policy

The authors declare that data supporting the findings of this study are available within the paper and its supplementary information files.

Human research participants

Policy information about studies involving human research participants and Sex and Gender in Research.

Reporting on sex and gender	n/a
Population characteristics	n/a
Recruitment	n/a
Ethics oversight	n/a

Note that full information on the approval of the study protocol must also be provided in the manuscript.

Field-specific reporting

Please select the one below that is the best fit for your research. If you are not sure, read the appropriate sections before making your selection.

☒ Life sciences ☐ Behavioural & social sciences ☐ Ecological, evolutionary & environmental sciences

For a reference copy of the document with all sections, see nature.com/documents/nr-reporting-summary-flat.pdf

Life sciences study design

All studies must disclose on these points even when the disclosure is negative.

Sample size	No statistical test was used to determine the sample size.
Data exclusions	No data were excluded from any of the analyses described.
Replication	All the experiments were performed once in multiple animal replicates and all the animal replicates are presented.
Randomization	Mice were randomly segregated into five treatment groups (or in seven treatment groups for the separate experiment RSVPreF3 vs RSV PostF). Cows were randomized into four treatment groups.
Blinding	All the experiments were conducted in an unblinded way since investigators were involved in overall conduct of the study.

Reporting for specific materials, systems and methods

We require information from authors about some types of materials, experimental systems and methods used in many studies. Here, indicate whether each material, system or method listed is relevant to your study. If you are not sure if a list item applies to your research, read the appropriate section before selecting a response.

Materials & experimental systems

n/a	Involved in the study	
☐	☒	Antibodies
☒	☐	Eukaryotic cell lines
☒	☐	Palaeontology and archaeology
☐	☒	Animals and other organisms
☒	☐	Clinical data
☒	☐	Dual use research of concern

Methods

n/a	Involved in the study	
☒	☐	ChIP-seq
☐	☒	Flow cytometry
☒	☐	MRI-based neuroimaging

Antibodies

Antibodies used	ICS: peptide pool stimulation done in presence of anti-CD28 (clone 37.51 for mice; clone L293 for cows), anti-CD49d (clone 9C10/MFR4.B for mice; clone L25 for cows). Incubated with: murine cells: anti-CD16/32 antibody, anti-CD4-V450, anti-CD8-PerCp-Cy5.5 antibodies; bovine cells: Alexa Fluor 647-conjugated mouse anti-bovine CD4; FITC-conjugated mouse anti-bovine CD8. Staining: mice: anti-IL2-FITC, anti-IFNγ-APC and anti-TNFα-PE; cattle: PE-conjugated mouse anti-bovine IFN-γ). All antibodies from BD Biosciences.
Validation	All antibodies were used as validated by the manufacturer, including titration on the relevant positive or negative cells.

Animals and other research organisms

Policy information about studies involving animals; ARRIVE guidelines recommended for reporting animal research, and Sex and Gender in Research

Laboratory animals	6–8 weeks old female CB6F1 mice purchased from Envigo, Horst, The Netherlands, and 3–9 year-old cows (Bos taurus) purchased from local farmers.
Wild animals	No wild animals were used in this study.
Reporting on sex	The study involved female mice and female cattle. Sex was not considered during the design or analysis of the experiments.
Field-collected samples	No field-collected samples were used in this study.
Ethics oversight	Husbandry/experiments were ethically reviewed and performed in accordance with Belgian and European laws/guidelines/policies for animal experimentation, housing, and care (Treaty ETS #123, Belgian Royal Decree 29-05-2013; European Directive 2010/63/EU), and GSK's Policy on the Care, Welfare, and Treatment of Animals. Protocols were approved by the local ethical review committees of GSK (mice: #P004/26/01; cows: #P00/000/00/Av1.00) and CER (cows; #CE/Santé/ET/012).

Note that full information on the approval of the study protocol must also be provided in the manuscript.

Flow Cytometry

Plots

Confirm that:

☒ The axis labels state the marker and fluorochrome used (e.g. CD4-FITC).

☒ The axis scales are clearly visible. Include numbers along axes only for bottom left plot of group (a 'group' is an analysis of identical markers).

☒ All plots are contour plots with outliers or pseudocolor plots.

☒ A numerical value for number of cells or percentage (with statistics) is provided.

Methodology

Sample preparation	Murine splenocytes and bovine peripheral blood mononuclear cells (PBMCs) were isolated from homogenized spleens and heparinized blood, respectively. For both models, cells were plated at 106 cells/well and stimulated with a pool of 15-mer peptides (1 µg/mL) overlapping by 11 amino acids covering the RSVPreF3 sequence, in the presence of anti-CD28 (clone 37.51 for mice; clone L293 for cows) and anti-CD49d (clone 9C10/MFR4.B for mice; clone L25 for cows) antibodies, or were left unstimulated (controls). Phorbol 12-myristate 13-acetate (PMA) ionomycine was used as positive control for the in vitro T-cell activation and stimulation of cytokine production. After 2 h at 37 °C, Brefeldin A was added for another 4 h. Plates were left overnight at 4 °C. Cells were centrifuged and resuspended in Flow Buffer (PBS 1×, 1% FCS). Murine cells were incubated at 4 °C, first for 10 min with anti-CD16/32 antibody, then for 30 min with anti-CD4-V450 and anti-CD8-PerCp-Cy5.5 antibodies and Live/Dead-PO (Invitrogen). Bovine PBMCs were incubated with Live/Dead Near-IR (Invitrogen; 30 min, room temperature), washed, stained (Alexa Fluor 647-conjugated mouse anti-bovine CD4; FITC-conjugated mouse anti-bovine CD8) for 30 min, centrifuged and washed. Splenocytes/PBMCs were fixed/permeabilized in 200 µL Cytofix-Cytoperm, incubated (20 min, 4 °C), washed in 1×Perm/Wash buffer, and stained for 1 h (mice) or 2 h (cattle) at 4 °C in 1×Perm/Wash buffer with antibodies (mice: anti-IL2-FITC, anti-IFNγ-APC and anti-TNFα-PE; cattle: PE-conjugated mouse anti-bovine IFN-γ). Cells were then washed twice with 1×Perm/Wash buffer in PBS.
Instrument	Flow cytometry data were acquired using LSR2 (mice) or LSRFortessa (cattle) flow cytometers.
Software	FlowJo software (Tree Star).
Cell population abundance	In all experiments, at least 20,000 events were acquired within the CD4+ lymphocyte population.
Gating strategy	Gating strategies of the mouse and bovine data are provided in Supplementary Fig. 2.

☒ Tick this box to confirm that a figure exemplifying the gating strategy is provided in the Supplementary Information.
